# Supplementary material for: Titanium Dioxide Nanoparticles Enhance Leakiness and Drug Permeability in Primary Human Hepatic Sinusoidal Endothelial Cells
Source: Int J Mol Sci. 2018 Dec 21;20(1):35. doi: 10.3390/ijms20010035 (PMC6337147; doi:10.3390/ijms20010035)
Supplement: Supplementary file 1 [file ijms-20-00035-s001.pdf]

Supplementary results

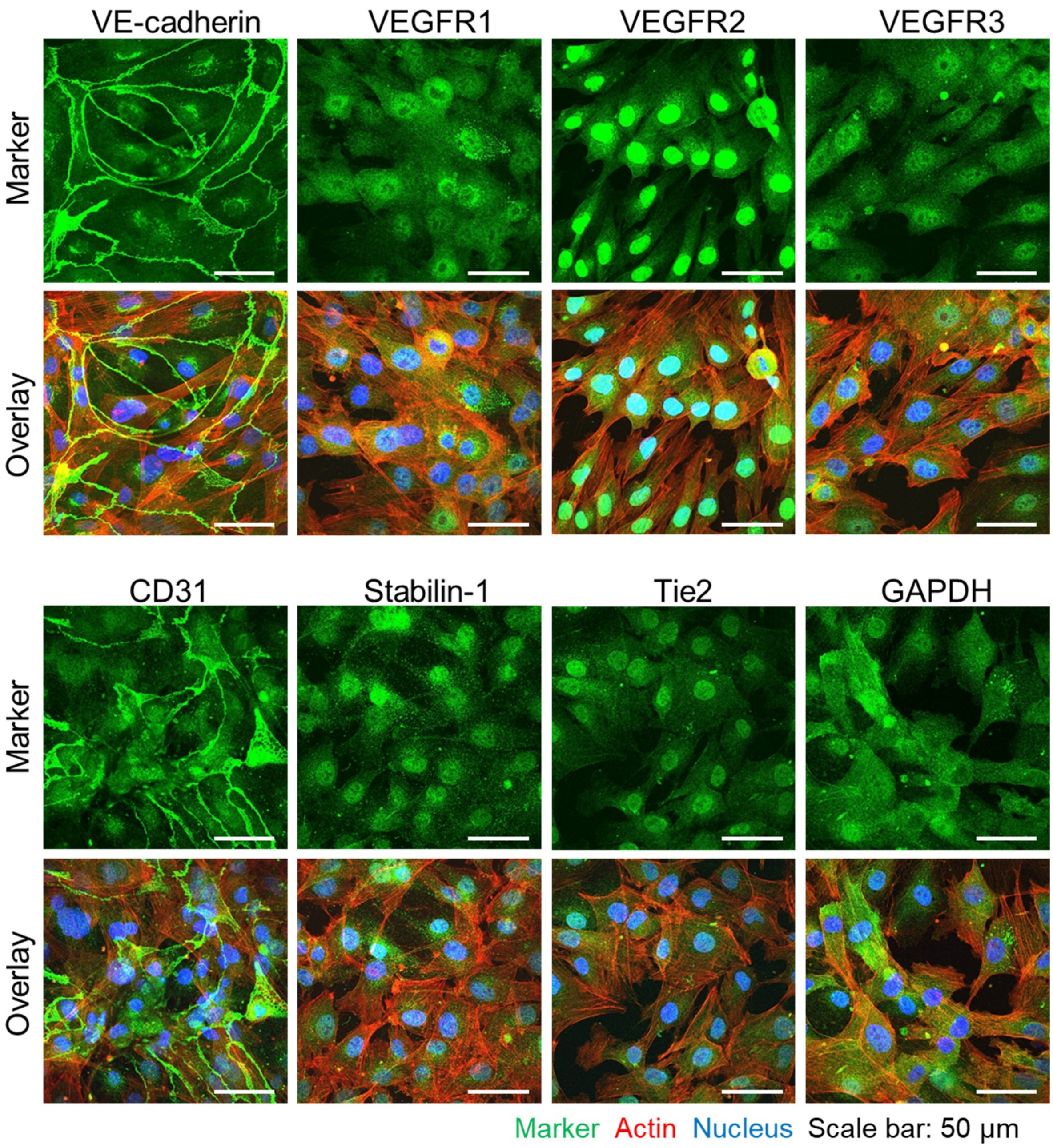

Figure S1. Characterization of biomarkers expressed in human hepatic sinusoidal endothelial cells (HHSECs). Immunofluorescence staining of HHSECs grown to confluence at 30,000 cells/cm^2 for 3 days. Protein of interest was stained with respective primary antibody followed by Alexa 488 conjugated antibody (green), actin was stained

with CF568 phalloidin dye and nucleus was stained with Hoechst dye (blue). Scale bar = 50 μm.

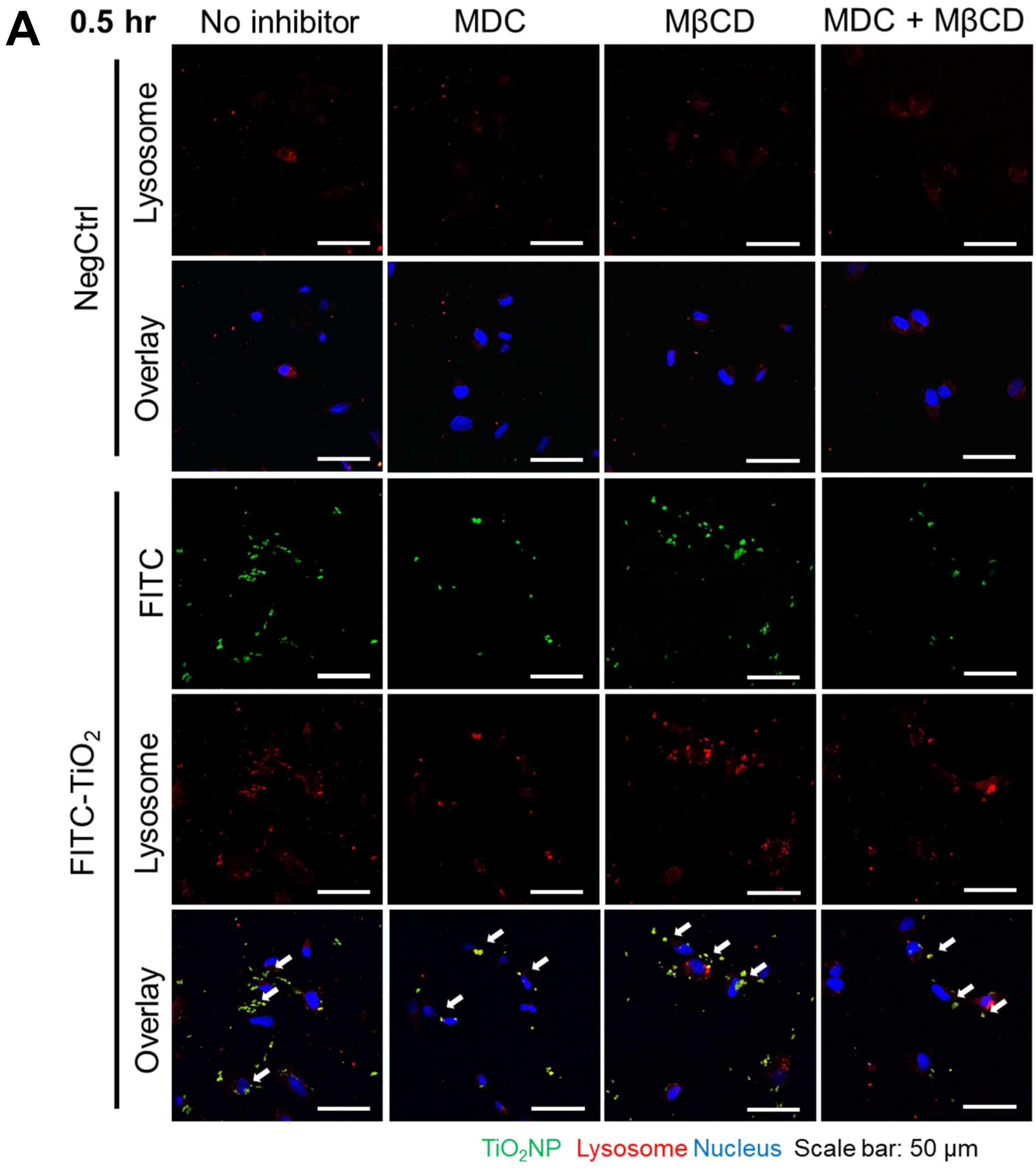

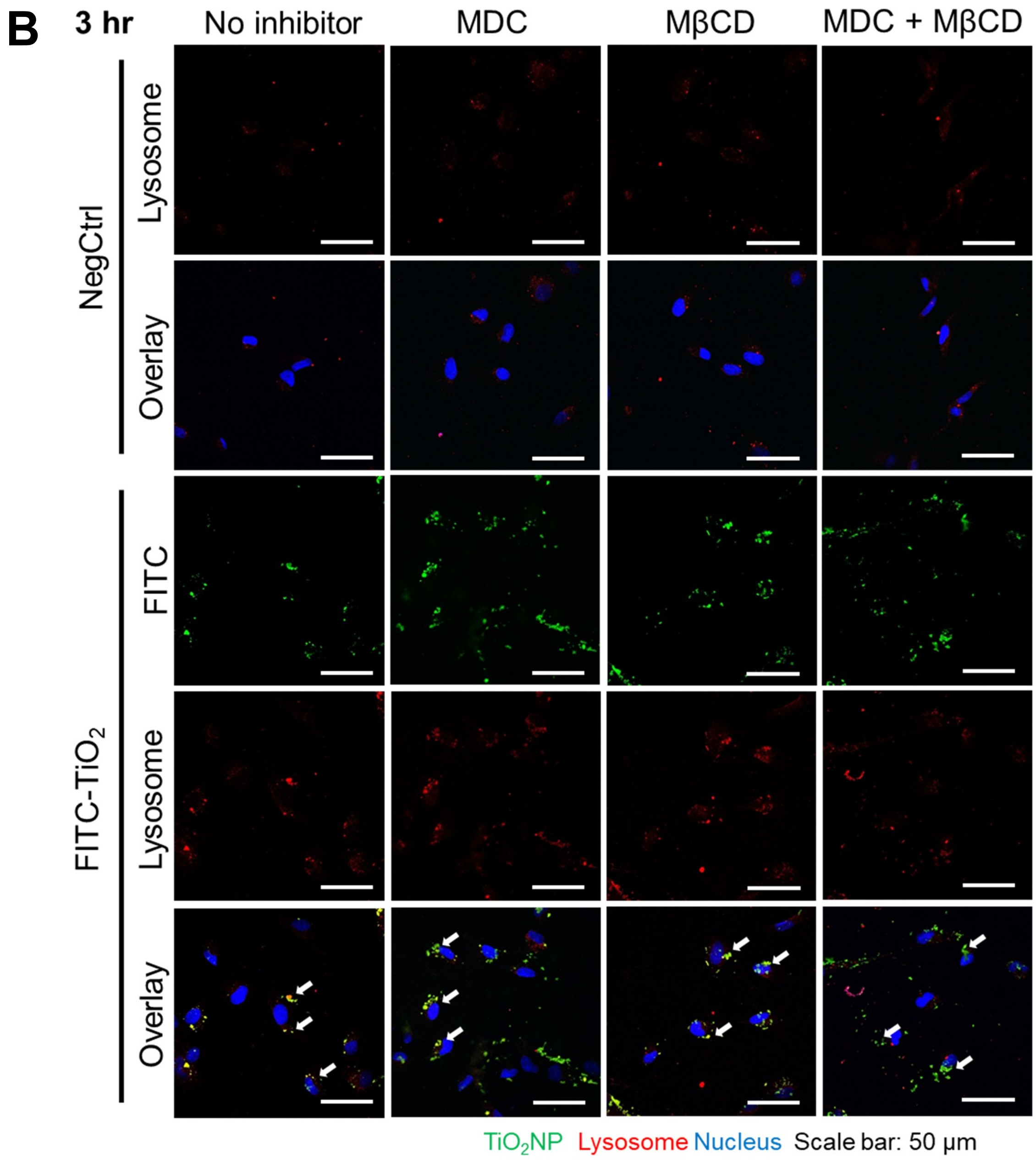

Figure S2. Endocytosis inhibitors did not completely inhibit the internalization of TiO_2 NPs into HHSECs. Cells were first pre-treated with the respective endocytosis

inhibitors for 15 mins before treatment of TiO_2 NPs conjugated with FITC (green) for (**A)** 0.5 hours and (**B**) 3 hours. Cells treated with or without inhibitors in the absence of NPs were used as the negative control (NegCtrl). Lysosome was stained with LysoTracker Red (red) and nucleus was stained with Hoechst dye (blue). Scale bar = 50 μm.

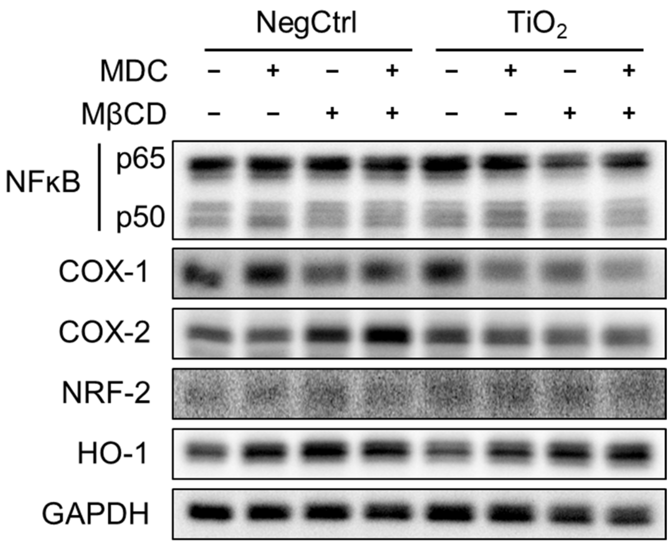

Figure S3. HHSECs treated with 100 μM TiO_2 NPs only for 3 hours in the absence or presence of endocytosis inhibitors did not show consistent increase in inflammation or oxidative stress markers. GAPDH was used as a loading control.

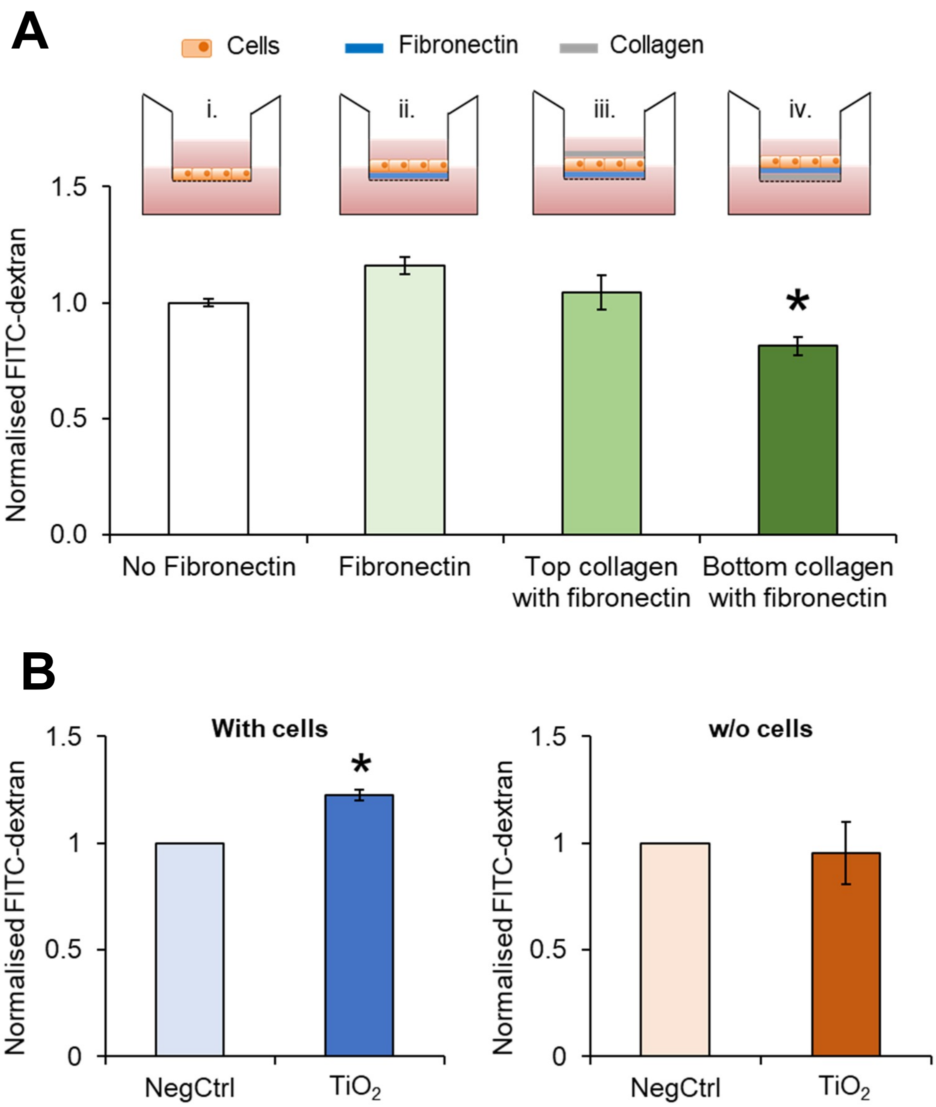

Figure S4. TiO_2 NPs induce endothelial leakiness in fibrotic HHSECs. (**A)** Optimization of fibrotic HHSEC model using (i) no coating, (ii) fibronectin only, (iii) collagen on top of cells with fibronectin and (iv) both coating below cells. Set-up (iv) exhibited reduced endothelial leakiness measured using 1 mg/ml FITC-dextran. Data represents mean ± SD, n = 3, *$p < 0.05$ (**B**) TiO_2 NP-induced leakiness was dependent on HHSECs seeded onto the transwell (left panel) whereas TiO_2 NPs did not result in leakiness of fibronectin and collagen coating in the absence of cells (right panel). Data represents mean ± SE, n = 5, *$p < 0.05$.
